# Fatty acid transport protein 1 regulates retinoid metabolism and photoreceptor development in mouse retina

**DOI:** 10.1371/journal.pone.0180148

**Published:** 2017-07-03

**Authors:** Aurélie Cubizolle, Laurent Guillou, Bertrand Mollereau, Christian P. Hamel, Philippe Brabet

**Affiliations:** 1 Inserm U1051, Institute for Neurosciences of Montpellier, Montpellier, France; 2 Laboratoire de Biologie et de Modélisation de la Cellule, Ecole Normale Supérieure de Lyon, Lyon, France; University of Florida, UNITED STATES

## Abstract

In retinal pigment epithelium (RPE), RPE65 catalyzes the isomerization of all-*trans*-retinyl fatty acid esters to 11-*cis*-retinol in the visual cycle and controls the rhodopsin regeneration rate. However, the mechanisms by which these processes are regulated are still unclear. Fatty Acid Transport Protein 1 (FATP1) is involved in fatty acid uptake and lipid metabolism in a variety of cell types. FATP1 co-localizes with RPE65 in RPE and inhibits its isomerase activity *in vitro*. Here, we further investigated the role of FATP1 in the visual cycle using transgenic mice that overexpress human FATP1 specifically in the RPE (hFATP1TG mice). The mice displayed no delay in the kinetics of regeneration of the visual chromophore 11-*cis*-retinal after photobleaching and had no defects in light sensitivity. However, the total retinoid content was higher in the hFATP1TG mice than in wild type mice, and the transgenic mice also displayed an age-related accumulation (up to 40%) of all-*trans*-retinal and retinyl esters that was not observed in control mice. Consistent with these results, hFATP1TG mice were more susceptible to light-induced photoreceptor degeneration. hFATP1 overexpression also induced an ~3.5-fold increase in retinosome autofluorescence, as measured by two-photon microscopy. Interestingly, hFATP1TG retina contained ~25% more photoreceptor cells and ~35% longer outer segments than wild type mice, revealing a non-cell-autonomous effect of hFATP1 expressed in the RPE. These data are the first to show that FATP1-mediated fatty acid uptake in the RPE controls both retinoid metabolism in the outer retina and photoreceptor development.

## Introduction

In the outer retina, the retinal pigment epithelium (RPE) is in close contact with rod and cone photoreceptors (PR); indeed, the apices of PR outer segments are engulfed in the apical microvilli of the RPE. This proximity is essential for the development, nutrition, detoxification, and survival of PRs, and RPE dysfunction can lead to pigmentary retinopathies and blindness [1]. The basal membrane of the RPE is in contact with the choroidal blood supply and can take up high concentrations of fatty acids and other metabolites [2].

The visual cycle, which is essential for sustaining vision, provides the 11-*cis*-retinaldehyde (11*c*RAL) chromophore to PR to regenerate visual pigments that sense light [3]. In mammals, photoactivation of rhodopsin in the rod outer segment (ROS) allows isomerization of 11*c*RAL to all-*trans*-retinal (a*t*RAL). On the cytoplasmic leaflet of disk membranes, a*t*RAL is reduced to all-*trans*-retinol (*at*ROL), which is then transported to the RPE. There, *at*ROL is esterified to all-*trans*-retinyl ester (a*t*RE) by addition of a long chain fatty acid (LCFA), most often palmitate, by a lecithin retinol acyltransferase (LRAT) [4]. RPE65 then isomerizes a*t*RE to 11-*cis*-retinol (11*c*ROL) [5] and releases the FA chain. Finally, 11*c*ROL is oxidized to form the chromophore 11*c*RAL by cis-retinol dehydrogenase.

RPE65 is highly abundant in the RPE but the RPE65-catalyzed isomerization is considered to be the rate-limiting step of the visual cycle [6]. Notably, Rpe65-deficient mice, which cannot synthesize 11*c*RAL and therefore lack functional rhodopsin [7], are protected against light-induced retinal degeneration [8]. The mechanisms responsible for the low activity of RPE65 are still poorly understood, but we [9] and others [10,11] have reported that RPE65 is negatively regulated. During aging, the visual cycle generates retinoid byproducts such as *N*-retinylidene-*N*-retinylethanolamine (A2E) that alter the RPE function and cause accumulation of lipofuscin, which, in turn, cause RPE and PR degeneration. This phenotype is well characterized in age-related macular degeneration (AMD) and Stargardt disease [12]. At present, there are no effective treatments that prevent loss of central vision in these diseases. Interestingly, mice with a slow visual cycle accumulate fewer cytotoxic byproducts [13], suggesting that the development of agents able to slow the production of deleterious retinal derivatives could be useful therapies for AMD and Stargardt disease.

In our previous study [9], we demonstrated that FATP1 is a negative regulator of RPE65 and acts by inhibiting its isomerase activity. In the mouse, Fatp1 and another member of the FATP family, Fatp4, are both expressed in the neural retina (NR) and RPE; in the NR, Fatp1 is mostly expressed in PRs [14]. Both proteins possess long and very long chain FA acyl-CoA synthetase activity [15,16]. Sequence analysis reveals that Fatp1 and Fatp4 are paralogs (>60% identity) and orthologs of the single *Drosophila fatp* gene. Since *fatp* is also required for PR survival and function, these findings suggest evolutionary conservation of FATP structure and function [17,18]. In support of this, we showed that *Fatp1*^-/-^ mice display only a small reduction in 11*c*RAL recovery after bleaching, suggesting that Fatp4 may have compensatory function. Similarly, although *Fatp1*^-/-^ mice exhibit accelerated aging of the outer retina, the visual cycle kinetics are normal [14]. More recently, FATP4 has also been shown to inhibit RPE65 [11]. Collectively, these findings suggested that FATP1 and FATP4 expressed in RPE may have overlapping functions.

To clarify the role of FATP1 in the retinoid cycle and vision, we investigated transgenic mice overexpressing human FATP1 (hFATP1TG) specifically in the RPE. We demonstrate not only that FATP1 overexpression regulates the content of retinoids involved in the visual cycle but also that FATP1 plays a non-cell-autonomous role in suppressing PR apoptosis during retinal development.

## Materials and methods

### Mice

Human FATP1 cDNA was cloned into the pcDNA3 vector downstream of the RPE-specific VMD2-585/+38 promoter (kindly provided by Noriko Esumi [19]). A 133 bp chimeric intron (Genebank U47119) (kindly provided by Anne Douar, Genethon, France) was introduced upstream of FATP1, and a bovine growth hormone poly (A) signal was added downstream of FATP1 to increase its expression. A PsiI-SalI 3950 bp fragment was purified and microinjected into C57BL/6N mouse embryos at SEAT transgenic animal service (UPS44 CNRS, Villejuif, France). Because C57BL/6N mice carry a *crb1* mutant allele, the transgenic founders were backcrossed to C57BL/6J (https://www.jax.org/strain/000664) until the mutant allele was removed. The hFATP1TG mice were bred at our institute in clear plastic cages and were subjected to standard light cycles of 12 h light (90 lux) and 12 h dark. The mice were fed *ad libitum* with a standard rodent diet. All animals were handled in strict accordance with the ARVO Statement for the Use of Animals in Ophthalmic Research and with EU directives. Animal care and use procedures conformed to French legislation (C34-172-36 from the Departmental Direction of Population Protection) and the Languedoc-Roussillon ethic committees (CEEA-LR-12141) approved the protocols.

### Electroretinography

All electrophysiological examinations were conducted using the VisioSystem (SIEM, France). Electroretinogram (ERG) recordings were performed with cotton electrodes as previously described [14]. For adaptation-ERGs, the mice were exposed to 7 repetitions of a 1.59 cd.s.m^−2^ blue flash, and the 7 b-wave amplitudes were averaged and taken as the dark-adapted control. The mice were then exposed to a 2 min photobleach at 300 lux, placed back in the dark, and subjected to the same series of flashes to verify abolition of the b-wave. This series of flashes was repeated every 4 min between 0 and 28 min to observe recovery of the b-wave.

### Retinoid quantification

Pupillary dilatation was induced by application of 0.5% tropicamide to the eyes (Mydriaticum, Thea, France). The mice were sacrificed by cervical dislocation before or at the indicated times after photobleaching (2,000 lux, 20 min). The eyes were rapidly enucleated, frozen in liquid nitrogen, and conserved at −80°C until use. Retinoids were extracted from eyes as described [14,20] with minor modifications. The eyes were homogenized in 800 μl of 3 M formaldehyde and incubated for 5 min at 30°C. Dichloromethane (1.5 ml) and hexane (3 ml) were successively added and the samples were centrifuged. The extracts were combined, evaporated and dissolved in 20 μl of ethanol for HPLC using a Varian HPLC system equipped with a C18 Isis column (4.6 × 250 mm) (Macherey-Nagel) and a Prostar 330 diode array detector. The retinoids were quantified from the peak areas using calibration curves determined with established standards.

### All-*trans*-retinal dehydrogenase (atRDH) activity

A*t*RDH activity was measured by monitoring the production of *at*ROL (reduction of *at*RAL) as described [21]. In brief, the 200 μl reaction mixture contained 100 μg protein from a NR homogenate prepared in PBS (pH 7.0) containing 1 mM *n*-dodecyl-β-maltoside and 1 mM NAD(P)H. The reaction was initiated by the addition of a*t*RAL (final concentration, 20 μM) and the samples were incubated at 37°C for various times. The reaction was terminated by the addition of 300 μl methanol, and retinoids were extracted in 3 ml of hexane and analyzed by HPLC using 10% diethyl ether in hexane.

### RNA extraction and quantitative PCR (qPCR)

Mice were euthanized by cervical dislocation and the eyes were enucleated and dissected. The NR was separated from the RPE-choroid, and the RPE-choroid was then scraped off the sclera. We chose to scrape off both tissues rather than to enzymatically separate the RPE alone to minimize the time and manipulation between death and tissue removal. Total RNA was isolated using an RNeasy Mini kit (Qiagen) according to the manufacturer’s protocol. Equivalent amounts of total RNA were used for first strand cDNA synthesis using a SuperScript III Reverse Transcriptase kit (Life Technologies). qPCR was performed using a LightCycler FastStart DNA Master PLUS SYBR Green I kit (Roche). Amplifications from NR and RPE-choroid RNA were normalized to actin and the RPE-specific gene Mertk, respectively, to avoid errors in quantification due to the presence of choroid RNA. Primers used for amplification were: Mertk: Fwd 5′-CAG TTT TAT CCT GAT GAG GAA GG-3′, Rev 5′-GAA GGC TGT GTT TCT GGT GAC-3′; β-actin Fwd 5′-GCT ACA GCT TCA CCA CCA CA-3′, Rev 5′-TCT CCA GGG AGG AAG AGG AT-3′; hFATP1: Fwd 5′-AGC CGC TTC TGG GAC GAC TG-3′, Rev 5′-CGT GAA CTC CTC CCA GAT GGC-3′; Bcl2L1: Fwd 5′-GAC AAG GAG ATG CAG GTA TTG G-3′, Rev 5′-TCC CGT AGA GAT CCA CAA AAG T-3′; Bax: Fwd 5′-TGA AGA CAG GGG CCT TTT TG-3′, Rev 5′-AAT TCG CCG GAG ACAC TCG-3′; mFatp4: Fwd 5′-CTG AAG CTG CCC TGG ACC CA-3′, Rev 5-AGG GCA TCC CGC CTA AGG TTG-3′; RPE65 Fwd 5′-GTG CCA CTG CTC ATC CAC ATA TTG-3′, Rev 5′-TGC AGG GGA ACT GCA CAA CAA CT-3′; LRAT: Fwd 5′-GAG CAG CAG TTG GGA CTG ACT-3′, Rev 5′-TCC CAA GAC AGC CGA AGC AAG A-3′; retinal dehydrogenase 5 (RDH5): Fwd 5′-TCA CCA GTG TCT TGG GCC GCA-3′, Rev 5′-AGG TTG GTC ACA GGG GTT CGA A-3′.

### Antibodies and western blotting

A rabbit polyclonal FATP1 antibody, produced using GST-FATP1c and purified as described [9], was used at 1:50 dilution. Other primary antibodies were mouse anti-rhodopsin (Novus Biologicals NBP1-47602, 1:500), mouse anti-α-tubulin (Sigma T5168, 1:1000), RPE65 (Novus Biologicals, 1:200), LRAT (Abcam ab73401, 1:200), RDH5 (Abnova HA00005959-A01, 1:500), and DGAT1 (Novus Biologicals NB110-41487, 1/500).

Tissues were lysed in SET buffer (10 mM Tris-HCl, pH 6.8, 1% SDS, 150 mM NaCl, 1 mM EDTA) plus protease inhibitors, homogenized, and centrifuged at 10,000 rpm for 3 min. Samples equivalent to 25 μg protein were separated by 10% SDS-PAGE (Mini-PROTEAN TGX gels, Bio-Rad) and electrotransferred to PVDF membranes (Trans-Blot Turbo Transfer System, Bio-Rad). After the membranes were blocked, they were incubated overnight at 4°C with the primary antibodies. The membranes were then washed, incubated with the appropriate horseradish peroxidase-conjugated secondary antibodies, and washed again. Finally, the membranes were developed using an enhanced chemiluminescence substrate (Pierce ECL, Thermo Scientific) and a V3 Western Workflow system (Bio-Rad). The protein bands were semi-quantified using densitometry and ImageJ software.

### Histology and hematoxylin-eosin-safranin (HES) staining

Animals were sacrificed by vertebral dislocation. The eyes were rapidly enucleated and fixed in 4% paraformaldehyde (PFA) for 24 h at 4°C. Eye cups were embedded in paraffin and cut into 5 μm sagittal sections. The sections were deparaffinized, stained with HES, rinsed, and mounted in Moviol.

### C_1_-Bodipy 500/510 C_12_ uptake

The eyes were rapidly enucleated and dissected in Hank’s balanced salt solution (HBSS). The cornea, the lens and the neural retina were removed to expose the RPE layer. The eyecup preparation was then flat-mounted as described with minors modifications [22]. The RPE was incubated in Hank’s balanced salt solution (HBSS) with 100 μg/ml of C_1_-Bodipy 500/510 C_12_ (Molecular Probes, D3823) at 37°C in the dark for various times, washed 3 times in HBSS, and fixed in 4% PFA for 15 min at room temperature in preparation for ZO-1 immunostaining.

### Immunofluorescence microscopy

Following C1-Bodipy 500/510 C12 labeling, the RPE was permeabilized with 0.1% SDS, blocked by incubation for 20 min with 10% fetal calf serum, and incubated overnight with a 1:500 dilution of rabbit anti-ZO-1 antibody (Invitrogen 40–2200). The RPE was washed and incubated for 4 h at room temperature (RT) with Alexa-647-conjugated anti-rabbit secondary antibody diluted in blocking buffer. The RPE was gently rinsed in PBS and labeled for 5 min with a 1:1000 dilution of 4′, 6-diamidino-2-phenylindole (DAPI), then rinsed 5 times in PBS for 5 min at RT and mounted in DAKO mounting medium. Confocal imaging of Bodipy C12/ZO-1 fluorescence was performed with a Zeiss LSM 5 LIVE DUO High-speed/Spectral Confocal system. Images were acquired using Zeiss Zen software.

### Light-induced retinal degeneration

Groups of mice (n = 3) were dark adapted for 48 h. The pupils were dilated by application of 0.5% tropicamide and the mice were exposed to bright light (20,000 lux) for 3 h in a white plastic bucket, after which they were kept in the dark for 5 days until euthanasia and histological analysis by HES staining.

### Ex vivo two-photon microscopy

Mice were dark adapted for 48 h and the pupils were dilated by application of 0.5% tropicamide. The mice were exposed to 20,000 lux for 15 min and then placed in the dark for 30 min before euthanasia by vertebral dislocation. The eyes were rapidly enucleated and dissected to expose the RPE layer in the eyecup, which was then flat mounted. ZO-1 immunofluorescence staining was performed as described above with the following modifications: the samples were incubated with primary anti-ZO-1 antibody for 2 h, the secondary antibody was Alexa-594-conjugated anti-rabbit antibody, and the DAPI labeling step was omitted. The flat-mounted RPE in PBS was analyzed by two-photon microscopy as previously described [23]. Retinosome autofluorescence and anti-ZO-1 fluorescence were observed with wavelengths of 780 nm and 1081 nm, respectively, using a Zeiss Confocal 7MP-OPO AxioExaminer. Images were acquired with the Zeiss Zen software.

### Spectral domain-optical coherence tomography (SD-OCT)

SD-OCT was performed separately on each eye of 6-month-old mice using an Envisu R2000 SD-OCT device (Bioptigen, Durham, NC). Mice were anesthetized by intraperitoneal injection of a mixture of ketamine (120 mg/kg) and xylasine (10 mg/kg) and the pupils were dilated by application of 1% tropicamide and 2.5% phenylephrine. Corneal hydration was maintained with Systane Ultra (Alcon, Fort Worth, TX) and Genteal (Novartis, Bale, Swiss) lubricant eye drops. Animal preparation and image acquisition were performed as previously described [24]. The analyses were performed using the rectangular scanning protocol (1.4 mm × 1.4 mm with 1000 A-scans per B-scan × 100 B-scans) while centered on the optic nerve. For analysis of segments, the thickness of the ONL was measured and expressed in arbitrary units. Ten measurements were performed per analysis and averaged for both eyes.

### Terminal deoxynucleotidyl transferase dUTP nick end labeling (TUNEL)

Animals were sacrificed by vertebral dislocation and the eyes were rapidly enucleated and fixed in 4% PFA for 24 h at 4°C. Eyecups were embedded in OCT compound and cut into 10 μm sagittal sections. TUNEL labeling was performed according to the manufacturer’s recommendations (Roche; In situ cell death detection kit, Fluorescein, 11684795910).

### Statistical analysis

All data are presented as means ± SEM and analyzed using the nonparametric Mann—Whitney test, with a significance threshold set at 5%, except those from the SD-OCT experiments (small sample size). For SD-OCT, the data were analyzed by two-way ANOVA followed by Bonferroni’s *post hoc* test. A p value of <0.05 was considered significant.

## Results

### Validation of hFATP1 overexpression in the RPE of hFATP1TG mice

**Fig 1:**
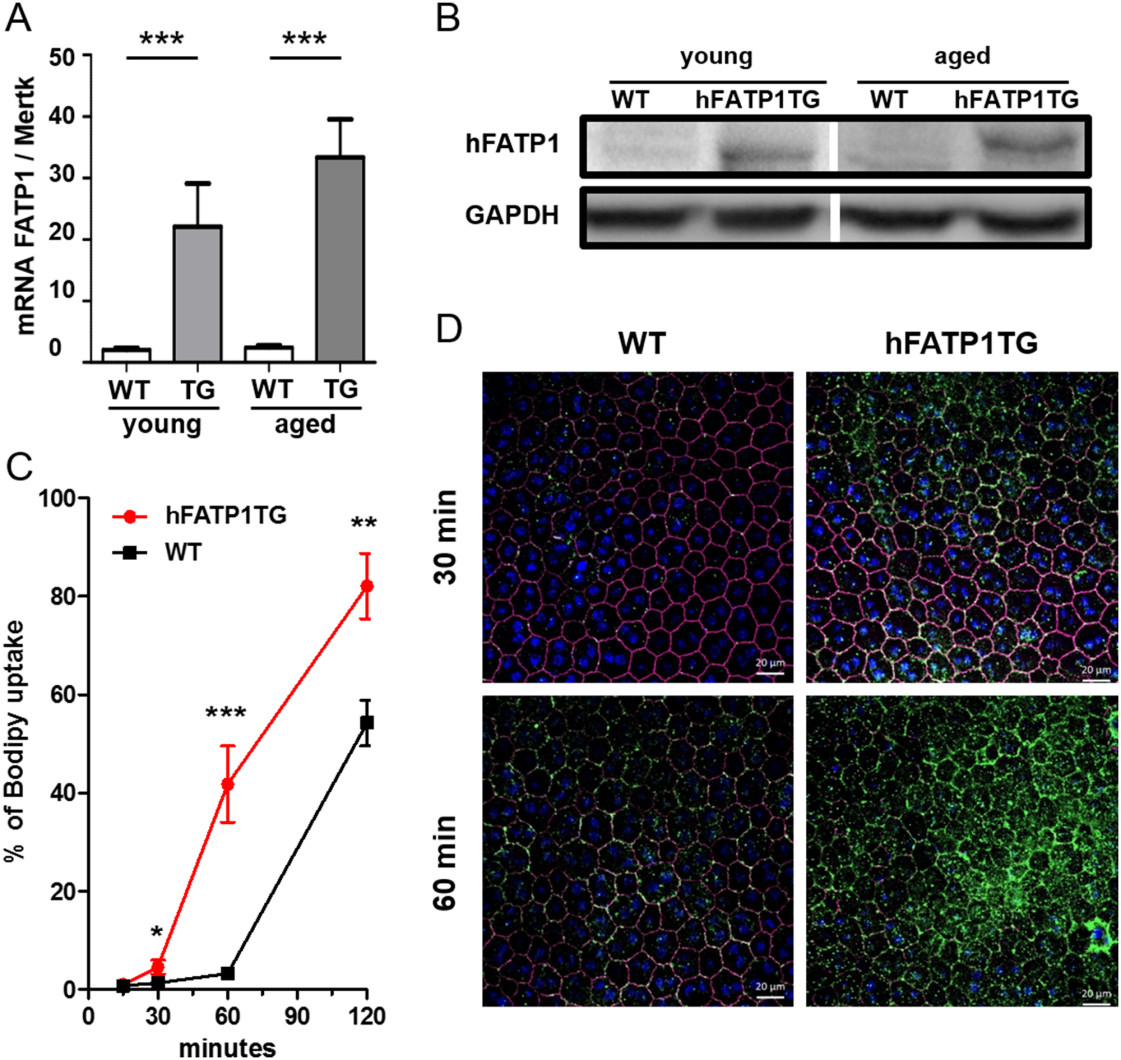
Validation of hFATP1 overexpression in the RPE of hFATP1TG mice. (A) qPCR analysis of FATP1 mRNA expression (human and mouse) in RPE of young (1–3-month-old, n = 4) and aged (6–9-month-old, n = 6) wild type (WT) and hFATP1 transgenic (TG) mice. Results are normalized to Mertk mRNA expression. (B) Western blot of hFATP1 protein expression in RPE of young and aged WT and hFATP1TG mice. GAPDH was probed as a loading control. (C) Kinetics of Bodipy C12 uptake, expressed as a percentage of total fluorescence, in flat-mounted RPE from young hFATP1TG and WT mice. The data are from n = 4–5 mice. *p < 0.05, **p < 0.01, ***p < 0.001. (D) Confocal fluorescence microscopy of Bodipy C12 uptake in flat mounts of RPE from young WT and hFATP1TG mice. DAPI and ZO-1 labeling permitted visualization of nuclei and tight junctions, respectively, of individual RPE cells.

We generated hFATP1 transgenic mice using the VMD2 promoter to drive selective overexpression in the RPE [19]. Expression of the hFATP1 transgene in the RPE-choroid fraction of eyes from young (1–3 months) and aged (6–12 months) mice was assessed (Fig 1). In Fig 1A, the level of mouse and human FATP1 mRNA combined was 11-fold higher in the young hFATP1TG mice (TG) than in age-matched wild type (WT) mice (22.08 ± 7.03 vs 2.03 ± 0.32; mean ± SEM normalized units) and 14-fold higher in aged hFATP1TG mice compared with aged WT mice (33.35 ± 6.18 vs 2.41 ± 0.37). Although the level of hFATP1 overexpression varied between individual transgenic mice, all of the animals showed significantly increased total (mouse and human) FATP1 mRNA levels. Overexpression of hFATP1 protein in the RPE of young and aged mice was confirmed by western blot analysis (Fig 1B). We examined the function of FATP1 in young hFATP1TG and WT mice by following the kinetics of uptake of Bodipy C12, a fluorescent FA acid analog, in *ex vivo* flat-mounted RPE (Fig 1C and 1D). The hFATP1TG RPE took up a significantly greater percentage of the total FA analog added than did the WT RPE at 1 h (41.8% ± 7.8 vs 3.2% ± 0.7) and 2 h (82.4% ± 6.65 vs 54.4% ± 4.6). FA uptake was also imaged by confocal microscopy of flat-mounted RPE. Co-labeling of RPE with Bodipy C12 and an antibody to the tight junction marker ZO-1 to delineate RPE65 cells revealed a striking difference in Bodipy C12 labeling in the hFATP1TG RPE compared with WT RPE (Fig 1D). Importantly, overexpression of hFATP1 in the mouse RPE had no effect on the endogenous expression of FATP4 in either the RPE or NR (S1 Fig). Collectively, these data validate the hFATP1TG mice as an excellent tool to investigate the function of FATP1 in the retina.

### Electroretinography of visual function in hFATP1TG mice

**Fig 2:**
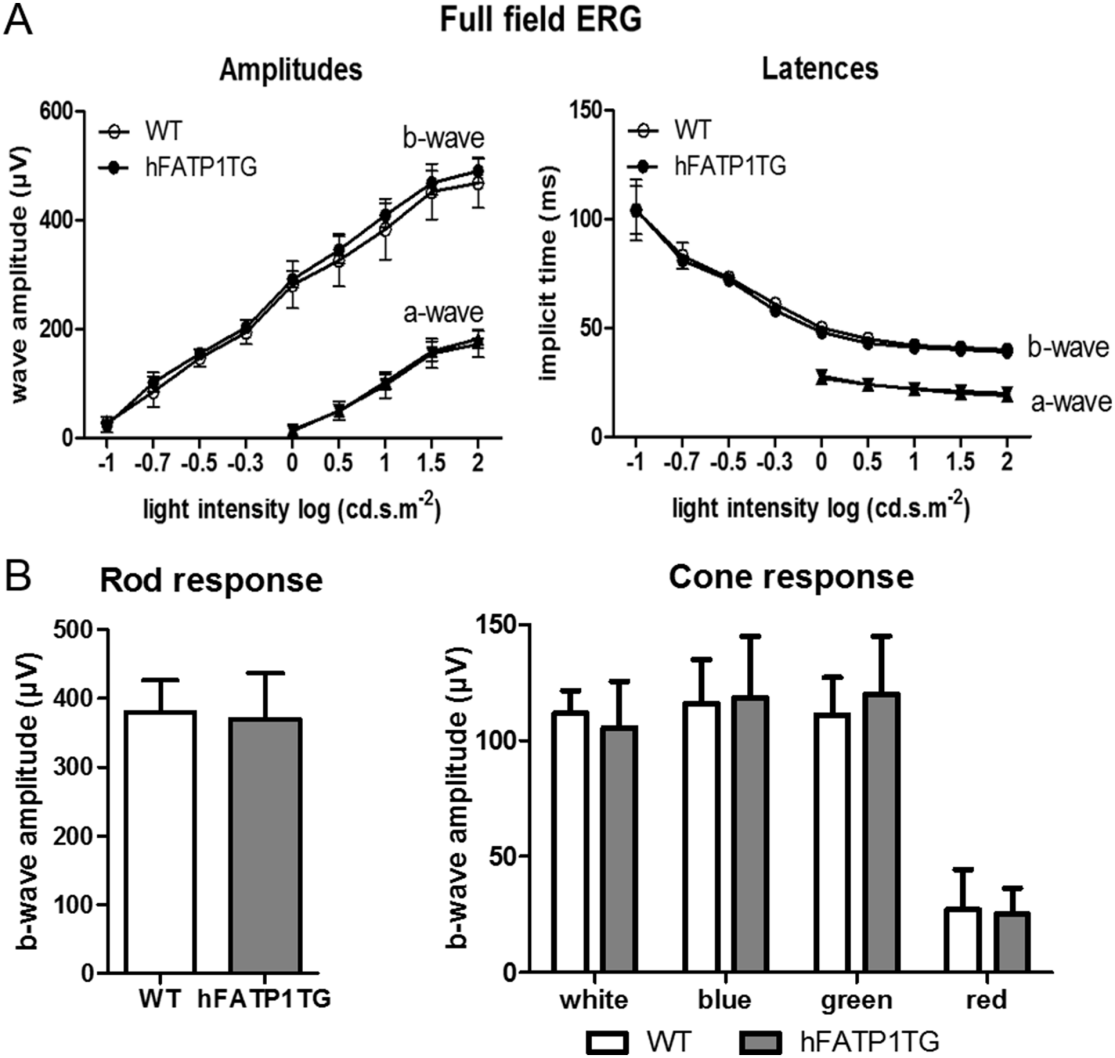
Visual function is not impaired by hFATP1 overexpression. (A) Full-field electroretinogram (ERG) analysis to determine light sensitivity of aged wild type (WT, n = 10) and hFATP1TG (n = 17) mice show no differences in a-wave and b-wave amplitudes or latencies. (B) Maximal b-wave amplitudes of rod and cone photoreceptors in aged WT (n = 6) and hFATP1TG (n = 8) mice. White light was used as stimulus for total rod and/or cone responses. Blue and green lights activate S and M cone photopigments respectively. No statistically significant differences were observed in any of the parameters measured.

We previously demonstrated *in vitro* that FATP1 can slow the visual cycle by inhibiting RPE65-mediated 11*c*ROL formation [9], suggesting that visual function may be disturbed in hFATP1TG mice. To assess this, we analyzed the electrical response of retinal cells to light stimulation by recording full-field ERGs (Fig 2). Mice were exposed to varying intensities of light, and the sensitivity to light intensity was measured by recording the amplitude and latency of the ERG responses for a-waves and b-waves, which reflect the activity of the outer layer PRs and the inner layer cells, respectively. We found no differences in any of these parameters between the young (not shown) and aged hFATP1TG and WT mice (Fig 2A), indicating that overexpression of hFATP1 had no effect on light sensitivity. We then analyzed the function of rods and cones separately by recording the ERG responses under scotopic conditions (rod response) and photopic conditions (cone response). Here, too, we detected no significant differences in the responses of hFATP1TG and WT mice (Fig 2B). Collectively, these results indicate that overexpression of hFATP1 did not impair visual function.

### Kinetics of chromophore regeneration

**Fig 3:**
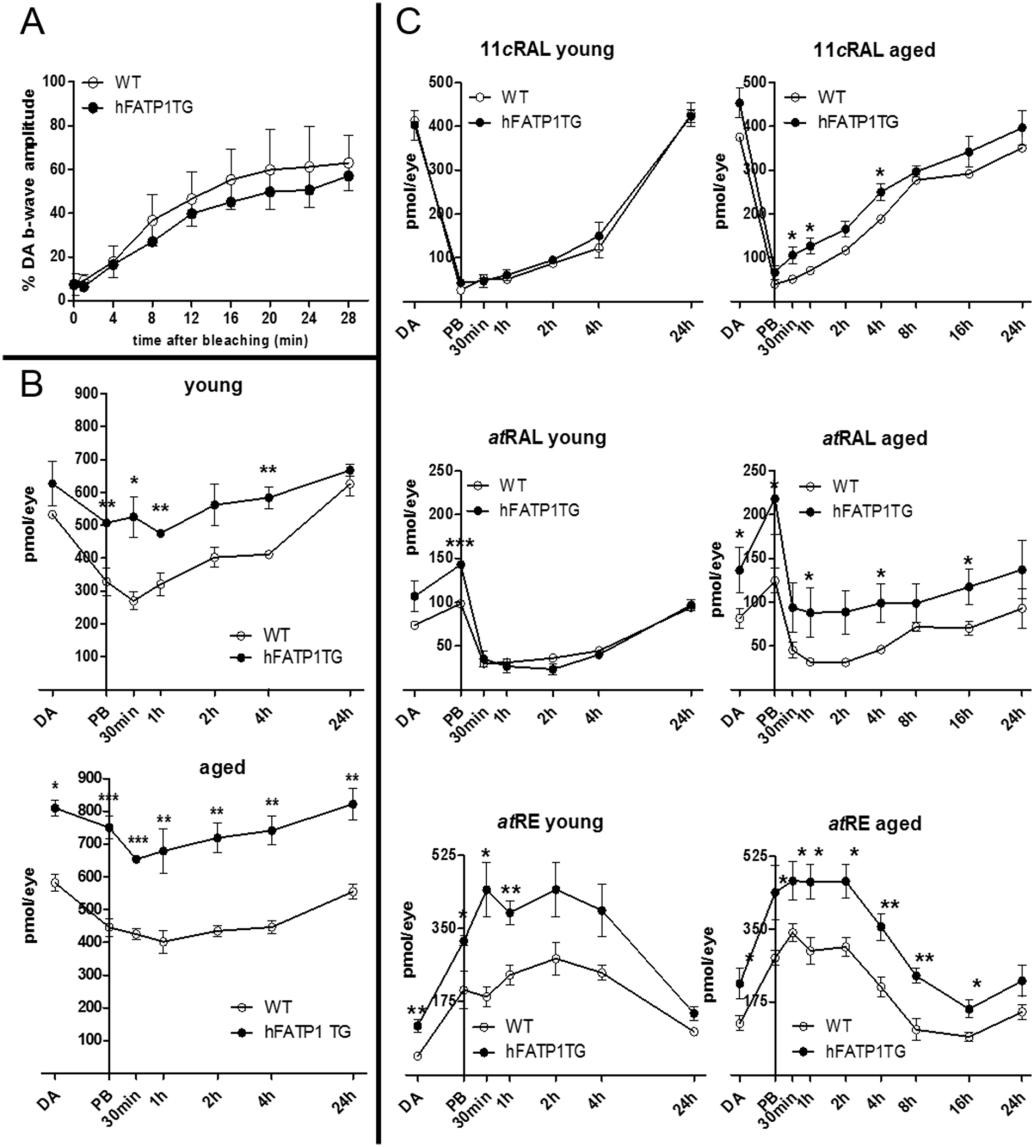
Kinetics of the recovery of retinoids during dark adaptation of hFATP1TG mice. (A) Kinetics of b-wave amplitude recovery in aged wild type (WT, n = 14) and hFATP1TG (n = 16) mice kept in darkness for the indicated times after photobleaching. Values are expressed as the percentage of the b-wave amplitude in dark-adapted (DA) mice. (B) HPLC quantification of total retinoids during dark adaptation of young (n = 3 per group) and aged (n = 6 per group) WT and hFATP1TG mice. (C) Kinetics of the recovery of 11*c*RAL (top), *at*RAL (middle), and *at*RE (bottom) contents in the retina of young (2-month-old, n = 3) and aged (4–6-month-old, n = 6) WT and hFATP1TG mice. Measurements were performed after overnight dark adaptation (DA), immediately after photobleaching (PB), or after being kept in darkness for the indicated times after PB. *p < 0.05, **p < 0.01, ***p < 0.001.

We next investigated the effects of hFATP1 overexpression on the visual cycle (Fig 3). First, we performed an analysis of the kinetics of visual chromophore regeneration by recording ERGs of dark-adapted aged hFATP1TG and WT mice. Mice were exposed to light and maintained in darkness for up to 28 min thereafter before measuring the percentage recovery of b-wave amplitude compared with dark-adapted mice (Fig 3A). Although we observed no significant differences between hFATP1TG and WT mice, there was a trend towards a slower recovery rate for the transgenic compared with WT mice. Next, we performed HPLC to quantify the total retinoid content of young (2 months) and aged (4–6 months) mice both before photobleaching and during the adaptation to darkness over for 24 h after photobleaching (Fig 3B). We observed a significantly higher retinoid content in young and aged transgenic mice compared with WT mice. In young mice, a 1.6 fold increase was observed during the first 4 hours of recovery in the dark. In aged mice, the retinoid content was constitutively 1.7 fold higher in transgenic than in WT. To investigate the differences in retinoid content in more detail, we measured the kinetics of 11*c*RAL, a*t*RAL, and a*t*RE regeneration before photobleaching and then after during adaptation in darkness (Fig 3C). The recovery rates of 11*c*RAL, a*t*RAL, and a*t*RE were unaffected by age in both the hFATP1TG and WT mice, but interestingly, the absolute quantity of a*t*RAL and, to a lesser extent, 11*c*RAL, was greater in the aged compared with young hFATP1TG mice. The levels of *at*RE were also higher in both young and aged transgenic mice than in WT mice. We examined the expression of key visual cycle enzymes in the RPE; namely, Lrat, Rpe65, and Rdh5, which are required for a*t*RE and 11*c*RAL synthesis from a*t*ROL. However, their expression was not significantly altered by overexpression of hFATP1 (S2 Fig). Thus, there was a specific age-related accumulation of total retinoids in hFATP1TG mice.

We also observed a clear effect of the transgene on the content of individual retinoid (Fig 3C). a*t*RE was present at significantly higher levels in dark-adapted young (2.6-fold) and aged (1.75-fold) mice compared with WT mice (young, 117 ± 16 vs 45 ± 9 pmol/eye; aged, 219 ± 36 vs 125 ± 18 pmol/eye). This difference was stable during dark adaptation, with an average increase in content of 1.8-fold for young and 1.75-fold for aged transgenic mice compared with WT mice.

In the young mice, the presence of the hFATP1 transgene increased a*t*RAL levels only during photoisomerization (PB: 98 ± 1 vs 143 ± 3 pmol/eye for WT and hFATP1TG mice, respectively; Fig 3C). Interestingly, *at*RAL levels remained high in the transgenic mice compared with WT mice during aging (DA: 82 ± 12 vs 137 ± 26 pmol/eye for WT and hFATP1TG mice, respectively). Moreover, a*t*RAL levels were higher in the hFATP1TG mice than WT mice throughout the dark adaptation period (average 2-fold higher; Fig 3C). 11*c*RAL content in young mice was unaffected by the presence of the transgene, whereas there was a significant accumulation of 11*c*RAL in the aged hFATP1TG mice during the first 4 h of dark adaptation (Fig 3C).

Collectively, these results emphasize that hFATP1 overexpression induces an increase in a*t*RE formation at a young age that is sustained with aging. In contrast, *at*RAL, and to a lesser extent 11*c*RAL, are only markedly affected by hFATP1 overexpression in aged mice.

### Accumulation of all-*trans* retinyl esters visualized by two-photon microscopy

**Fig 4:**
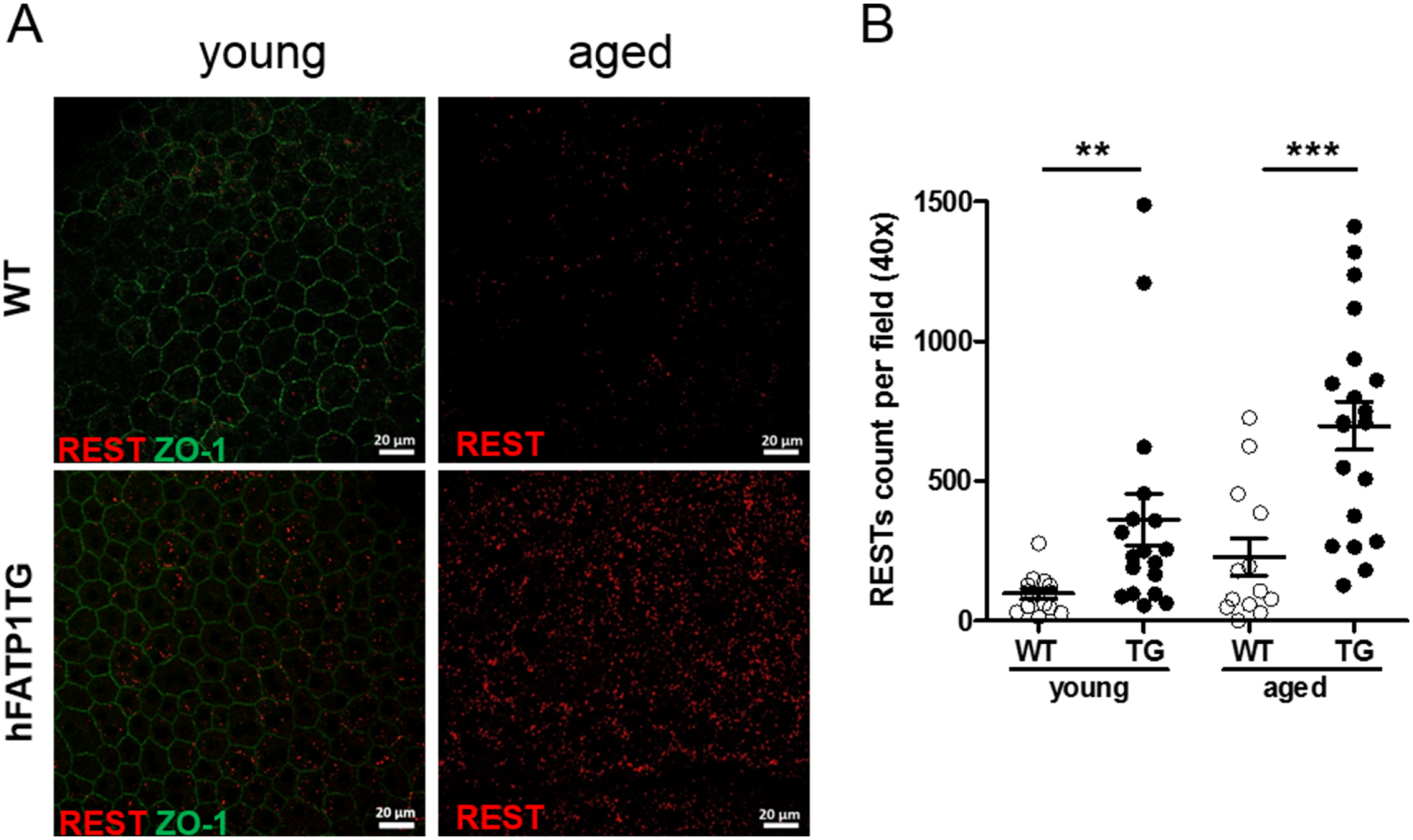
Age-related accumulation of retinosomes in the RPE of hFATP1TG mice. (A) Two-photon microscopy (wavelength 780 nm) of retinosome (REST) autofluorescence (red) in whole flat mount preparations of RPE. Immunostaining of tight junctions with anti-ZO-1 (green) delineates RPE cells. (B) Quantification of autofluorescent RESTs in RPE of young and aged WT and hFATP1TG (hF1TG) mice (n = 3–4). Means± SEM are calculated from 13–20 fields. **p < 0.01, ***p < 0.001.

Retinyl esters are stored in RPE lipid droplets called retinosomes or RESTs. To confirm the accumulation of a*t*RE in hFATP1TG mice observed by HPLC quantification, we performed two-photon microscopy to visualize RESTs as autofluorescent foci (Fig 4). To delineate individual cells, flat-mounted RPE were also stained with an antibody to ZO-1, and images were acquired during excitation at 780 nm (autofluorescent RESTs) or 1081 nm for ZO-1. This analysis clearly showed that RESTs were much more abundant in the RPE of hFATP1TG mice compared with WT mice, and this was particularly pronounced in the aged animals (Fig 4A). Quantification of the foci using Image J software (Fig 4B) revealed 3–4-fold more RESTs in the transgenic mice than in WT mice (young: 362.4 ± 92.0 vs 97.3 ± 18.4 foci per field; aged: 698.2 ± 85.5 vs 228.7 ± 66.8 foci per field). These data demonstrate that a*t*RE are specifically stored in REST in a regulated manner as dictated by hFATP1 overexpression.

### Light-induced photoreceptor degeneration

**Fig 5:**
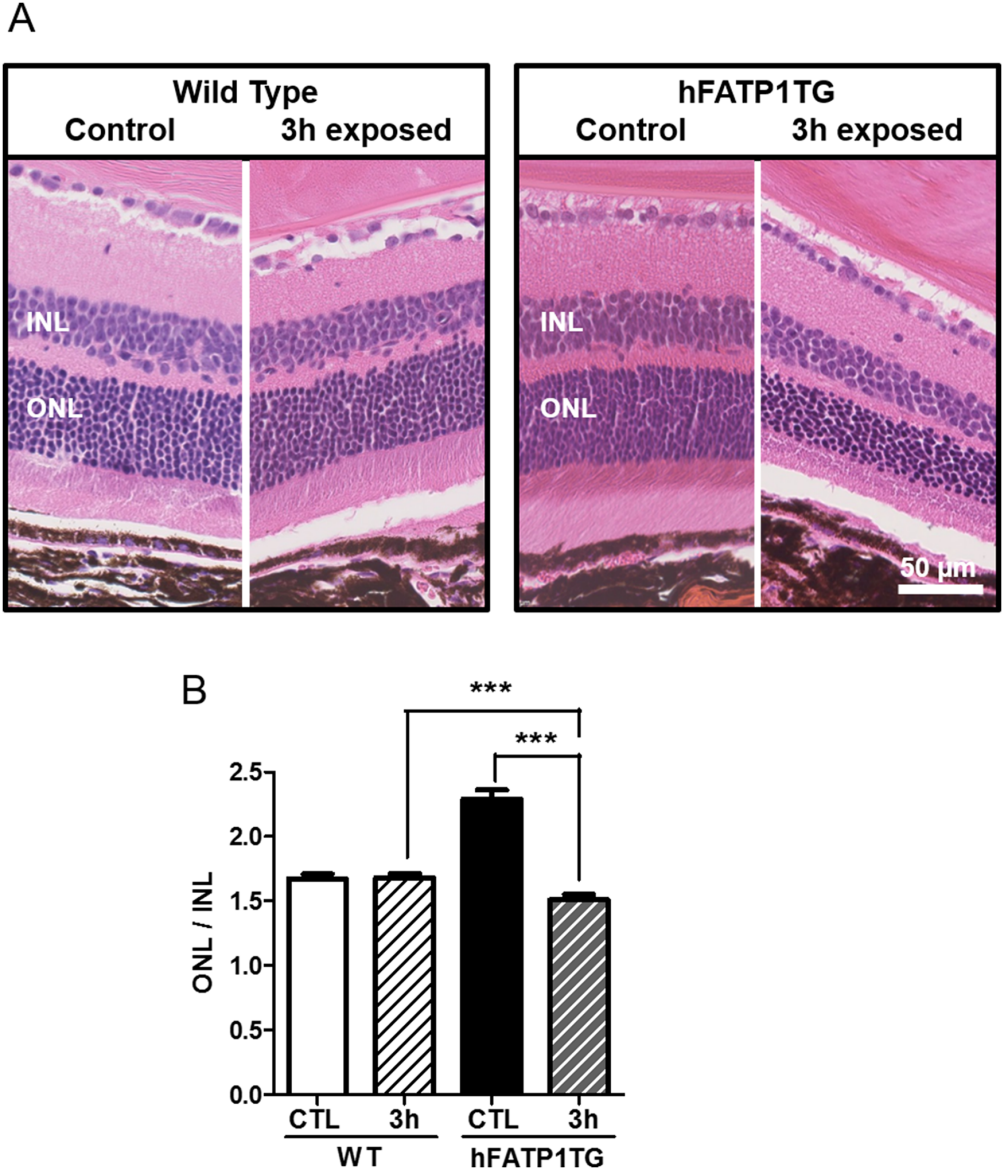
Light-induced retinal degeneration in hFATP1TG mice. (A) H&E and safranin staining of the retinas of aged wild type (WT) and hFATP1TG mice (n = 3 per group) after dark adaptation (control) or bright light exposure (3 h, 20,000 lux) followed by darkness for 5 days. (B) Quantification of photoreceptor loss measured as the ratio of outer and inner nuclear layer thickness (ONL/INL) under the conditions shown in (A). The INL thickness did not change significantly with age and was used as the reference. ***p < 0.001.

The accumulation of a*t*RAL in the PRs of aged hFATP1TG mice could contribute to an increased susceptibility to light-induced retinal damage. To test this hypothesis, aged hFATP1TG and WT mice were exposed to white fluorescent light for 3 h and then kept in darkness for 5 days before histological analysis of the retina (Fig 5). In the WT mice, the structure and thickness of the outer nuclear layer (ONL, rod and cone bodies) and the inner nuclear layer (horizontal, bipolar, and amacrine cells) was unaffected by light exposure (Fig 5A, left panel), indicating a resistance to light damage. In contrast, the hFATP1TG mice showed a marked loss of PR cells (Fig 5A, right panel). The ratio of ONL/INL thickness was used to quantify this loss since INL thickness was unchanged by light exposure in either genotype. Indeed, the ONL/INL ratio was significantly smaller in light-exposed hFATP1TG mice than in similarly treated WT mice (Fig 5B). We conclude that hFATP1 overexpression in the RPE promotes light-induced PR degeneration.

### Non-cell-autonomous effect of hFATP1 in the neural retina

**Fig 6:**
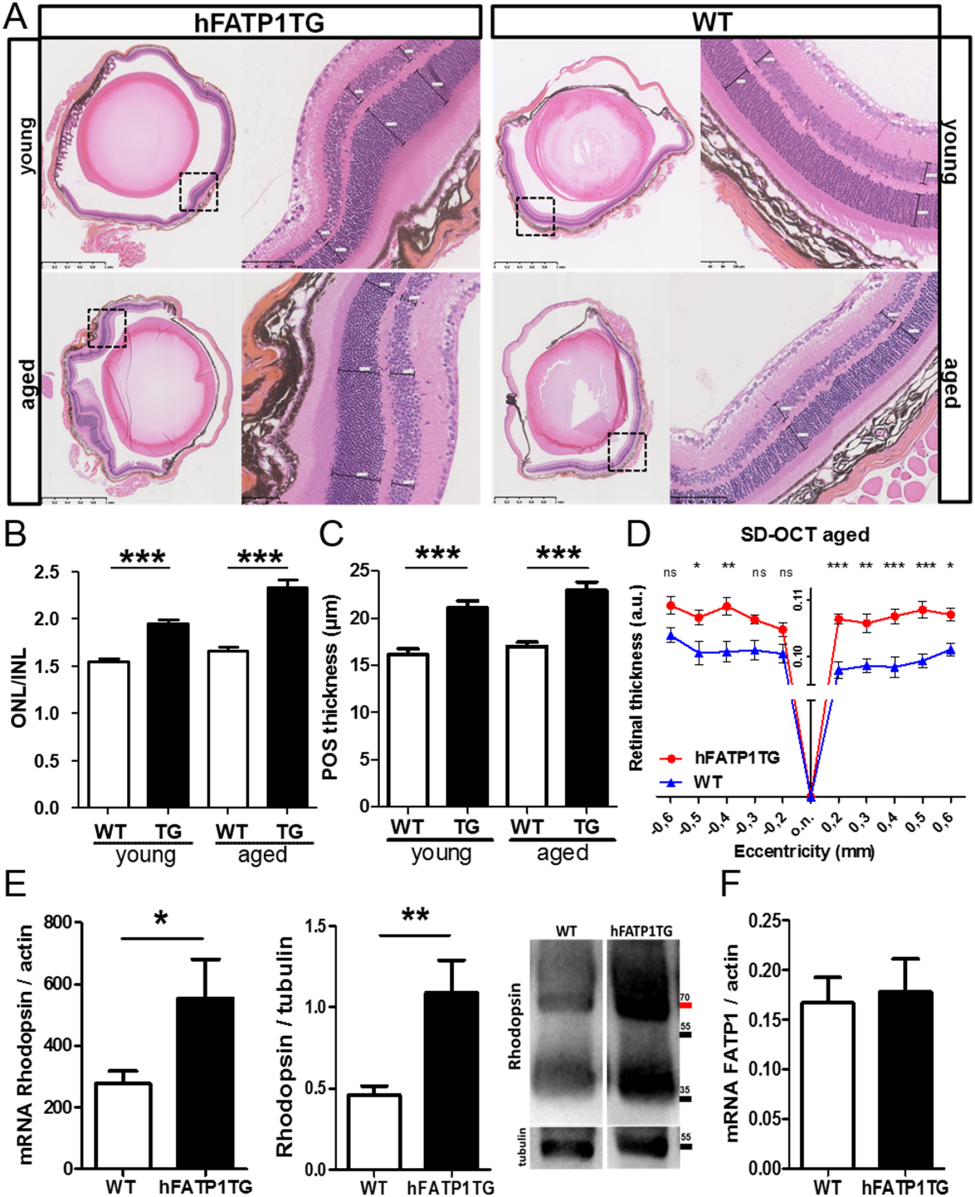
Non-cell-autonomous effects of hFATP1 in the neural retina. (A) H&E and safranin staining of 5 μm sagittal sections of eyecups of young (3-month-old) and aged (6–15-month-old) hFATP1TG and wild type (WT) mice. (B) Quantification of the ratio of outer and inner nuclear layer thickness (ONL/INL) of young and aged WT and transgenic (TG) and mice (n = 20 sections per mouse, 5 mice per group). (C) Quantification of photoreceptor outer segment (POS) length in young and aged WT mice and transgenic (TG) (n = 20 measures of length throughout the retina per mouse, 5 mice per group). (D) Spectral domain-optical coherence tomography (SD-OCT) measurements of retinal thickness of aged hFATP1TG and WT mice (n = 4 per group). o.n., optic nerve. (E) Quantification of rhodopsin mRNA (qPCR) and protein (western blot) levels in the neural retina of WT (n = 7) and hFATP1TG (n = 8) mice. (F) qPCR quantification of FATP1 mRNA levels in the neural retina of WT (n = 8) and transgenic (n = 7) mice. Data were normalized to actin mRNA and tubulin protein levels. *p < 0.05, **p < 0.01, ***p < 0.001.

To further understand the impact of high retinoid levels in the RPE on retinal function, we analyzed the NR morphology in young and aged mice by HES staining (Fig 6). In the hFATP1TG mice of both ages, we observed an irregular enlargement of discrete regions of the NR that was not apparent in the WT mice (Fig 6A). Moreover, quantification of PRs by measuring the ONL/INL ratio showed that hFATP1 overexpression significantly increased the ONL (Fig 6B). In young animals, the ONL/INL ratio was 1.95 ± 0.05 and 1.55 ± 0.03 for hFATP1TG and WT mice, respectively. In aged mice, this ratio was 2.36 ± 0.13 for the transgenic mice and 1.60 ± 0.07 for the WT mice. We also measured the length of the PR outer segment (POS) and found that it was significantly longer in hFATP1TG mice than in WT mice (Fig 6C). Consistent with the enlargement of the PR layer, the global thickness of the retina was larger in hFATP1TG mice, as measured by spectral domain-optical coherence tomography (SD-OCT, Fig 6D) at 0.107 ± 0.001 and 0.091 ± 0.001 arbitrary units for the aged hFATP1TG and WT mice, respectively. Finally, we measured the expression of rhodopsin mRNA and protein levels in the NR and found that they were 2.4-fold and 2-fold higher, respectively, in the hFATP1TG mice than the WT mice (Fig 6E). As expected, given the use of an RPE-specific promotor, qPCR analysis showed no overexpression of hFATP1 in the NR (Fig 6F). Collectively, these results demonstrate that the density of PR was increased by hFATP1 overexpression in RPE.

**Fig 7:**
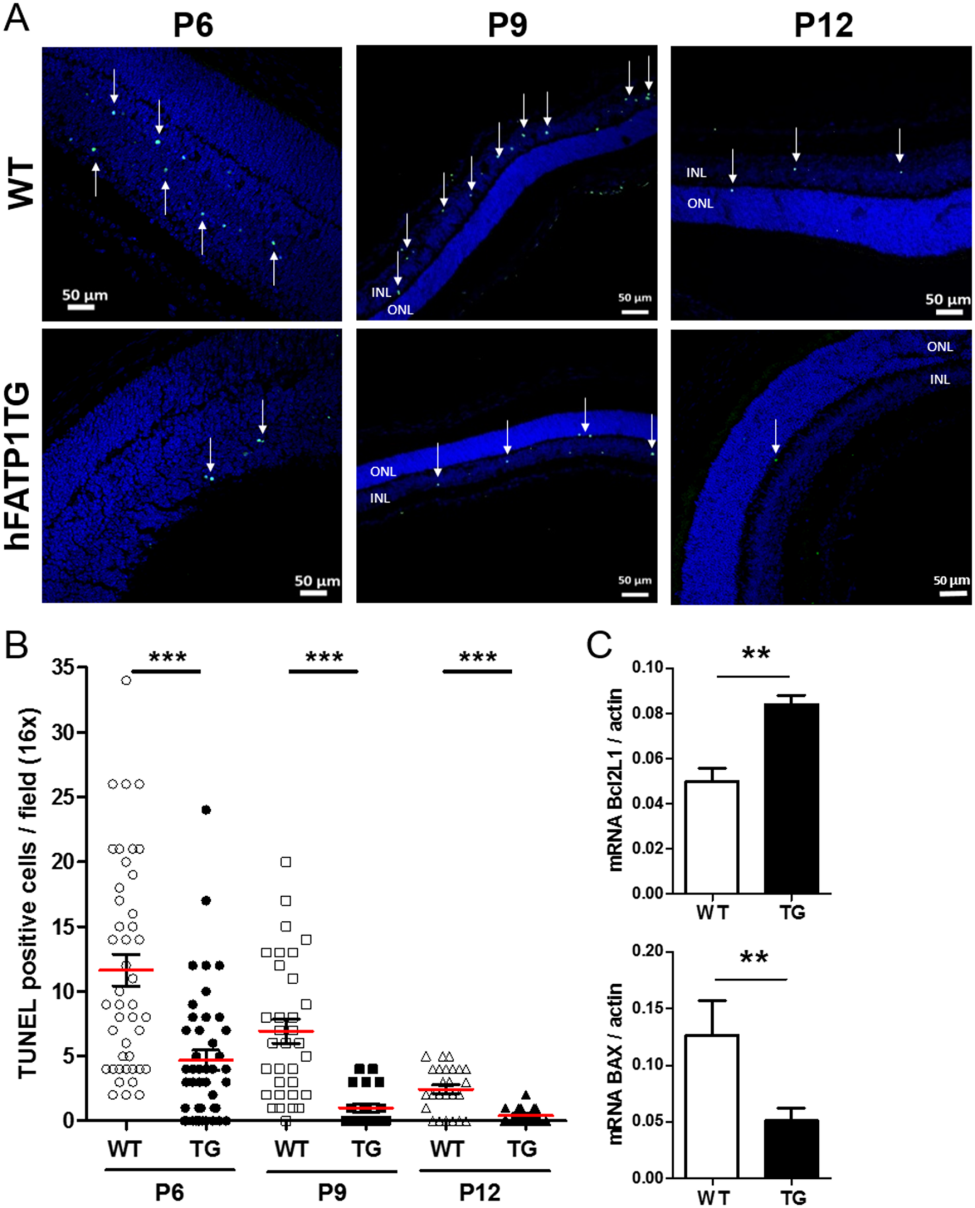
Non-cell-autonomous effects of hFATP1 on photoreceptor development. (A) TUNEL labeling (green foci, indicated by white arrows) in the retina of hFATP1TG and wild type (WT) mice on postnatal (P) days 6, 9, and 12 (n = 5 mice per group). (B) Quantification of TUNEL positive cells in the retina of P6, P9, and P12 transgenic (F1TG) and WT mice. TUNEL-positive cells were counted in all regions of the retina and are expressed as the number per field at 16× magnification (n = 5 mice per group). The results reveal a high frequency of apoptosis at P6 and P9, which is suppressed by hFATP1 overexpression. Mean values are represented by red bars. (C) qPCR quantification of Bcl2L1 and BAX mRNA in the neural retina of young hFATP1TG (hF1TG, n = 7) and wild type (WT, n = 6) mice. mRNA levels were normalized with actin.**p < 0.01, ***p < 0.001.

Given the continuous exchange of materials, including fatty acids, between the RPE and PRs, we hypothesized that the higher number of PRs in the transgenic mice might be due to an effect of RPE-expressed hFATP1 on the development of the NR. Since previous studies have reported an antiapoptotic role for FATP1 [25], we speculated that hFATP1 might inhibit the wave of PR apoptosis that occurs during NR development. To assess our hypothesis, we used the TUNEL assay to analyze the number of apoptotic cells in the NR of transgenic and WT mice on postnatal (P) days 6, 9, and 12 (Fig 7). Imaging of the retinas showed fewer TUNEL-positive cells in the hFATP1TG retinas than the WT retinas at P6, P9, and P12 (Fig 7A), and this was confirmed by quantification of the apoptotic cells (Fig 7B). At P6, hFATP1TG retinas contained 2.5-fold fewer apoptotic cells (4.69 ± 0.75 vs 11.6 ± 1.2). At P9, there were 7-fold fewer (vs 1.0 ± 0.3 vs 6.90 ± 0.92), and at P12, there were 6-fold fewer (2.44 ± 0.35 vs 0.41 ± 0.15) (Fig 7B), supporting the possibility that hFATP1 overexpression prevented PR apoptosis. Finally, we performed qPCR analysis to quantify mRNA levels of the antiapoptotic gene Bcl2L1 and the proapoptotic gene BAX (Fig 7C). The RPE of hFATP1TG mice expressed 1.7-fold higher levels of Bcl2L1 mRNA (0.085 ± 0.004 vs 0.05 ± 0.006 normalized units) and 4.3-fold lower levels of BAX mRNA (0.05 ± 0.01 vs 0.13 ± 0.03 normalized units; Fig 7C). These results support the notion that FATP1 plays an antiapoptotic role in NR development, and provide an explanation for the higher number of PRs in the transgenic mice.

## Discussion

Here, we examined the role of FATP1 in the visual cycle using a transgenic mouse model in which hFATP1 expression was driven by an RPE-selective promoter. hFATP1 was stably and specifically expressed in RPE cells, without detectable compensatory effects on the expression of FATP4 or other genes involved in retinoid metabolism. Moreover, we detected no ectopic expression of hFATP1 in the NR. Previous work has shown that the pattern of transgene expression in the RPE driven by the VMD2 promoter can be patchy and mosaic [19,26]. Consistent with such discontinuous expression, we found that enlargement of the PR layer in hFATP1TG mice was not homogeneous but occurred in discrete regions of the retina.

**Fig 8:**
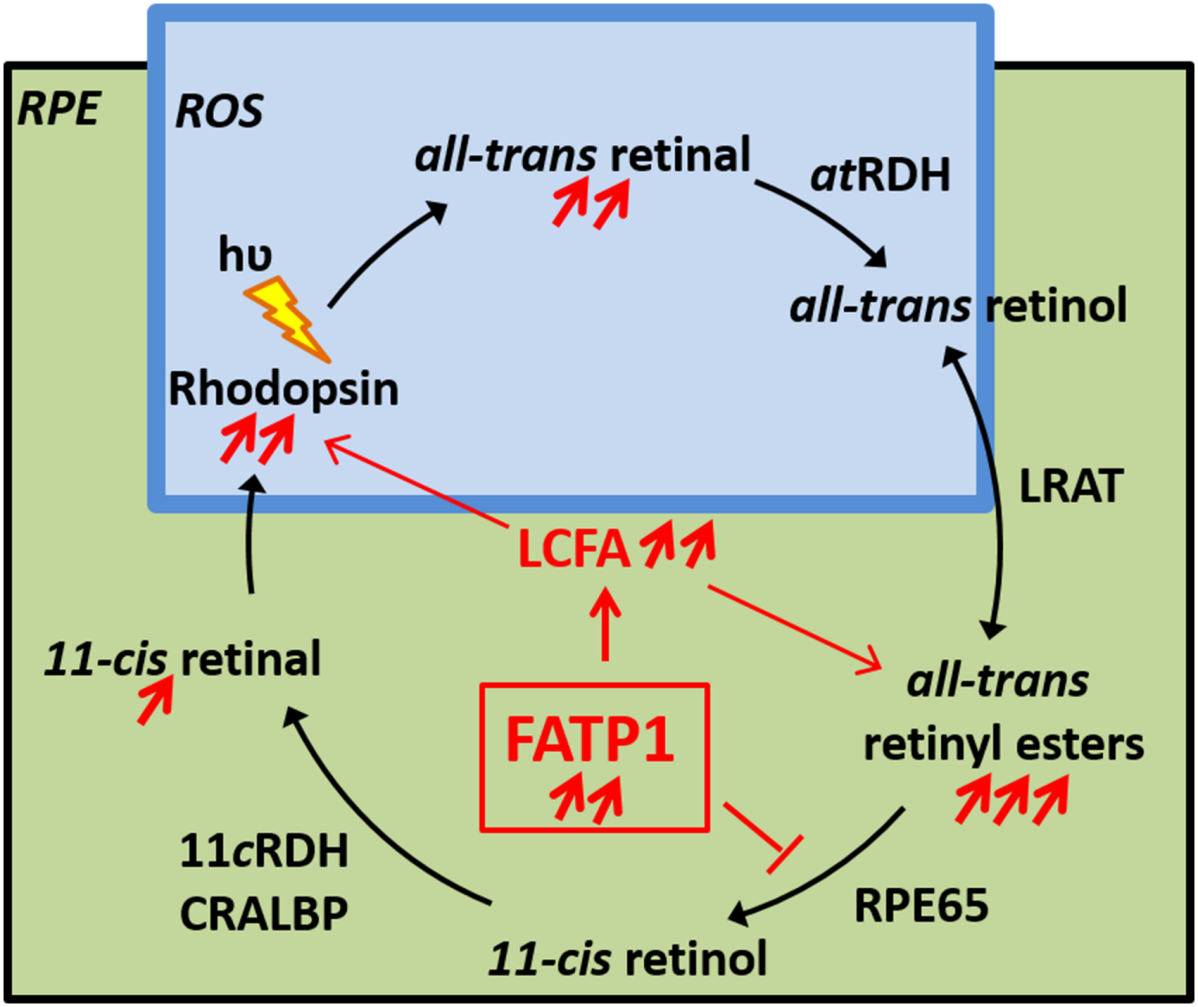
Regulation of the visual retinoid cycle by FATP1. Schematic summarizing the effects of FATP1 overexpression in the mouse RPE. Rhodopsin, the light-sensitive protein in rods, is located in the disk membranes of rod outer segments (ROS). Absorption of a photon (hʋ) induces 11-*cis* to all-*trans* isomerization of retinaldehyde. All-*trans*-retinal then dissociates from rhodopsin and is reduced to all-*trans*-retinol, which is taken up by an RPE cell. Also in the RPE, FATP1 promotes long chain fatty acid (LCFA) uptake, thus providing LCFA-CoA for formation of phospholipids. All-*trans*-retinol is esterified with a phosphatidylcholine (PC)-derived LCFA to form all-*trans*-retinyl esters in a reaction catalyzed by LRAT. RPE65 then converts all-*trans*-retinyl esters to 11-*cis*-retinol. All-trans retinyl esters accumulate in the transgenic hFATP1TG RPE, suggesting increased levels of LCFAs and/or inhibition of RPE65. In ROS, the response to light remains unchanged, although rhodopsin expression increases. This increase is related to a greater number of PRs and their longer outer segments. Consequently, the rate of all-*trans* retinal formation is elevated, resulting in susceptibility to degeneration induced by light.

We examined the effects of aging on retinal ERG responses and retinoid content in hFATP1TG mice. Our data show that neither the light sensitivity recovery rate nor the chromophore regeneration rate were changed in the hFATP1TG mice compared with the WT mice, suggesting that the visual cycle kinetics are unaffected by alterations in FATP1 levels. However, the retinal content of a*t*RE was significantly higher in young hFATP1TG mice than WT mice and the content of both a*t*RE and a*t*RAL increased with age. By contrast, 11*c*RAL regeneration was unchanged. These data suggest that the roles of FATP1 in both the uptake of LCFA and the inhibition of RPE65 likely contribute to the imbalance between the high rate of a*t*RE regeneration and the unchanged regeneration of 11*c*RAL in the RPE. Fig 8 summarizes our working model to explain our findings in the context of visual cycle regulation by FATP1.

The high levels of a*t*RE we observed in the RPE of hFATP1TG mice could be a direct effect of hFATP1 in enhancing palmitate uptake and palmitate-CoA generation [27], which in turn may contribute to phosphatidylcholine (lecithin) synthesis. FATP1 is an acyl-CoA synthase (ACS) that activates LCFAs and cooperates with additional ACS proteins in the process of vectorial acylation of LCFAs [28,29]. Intracellular esterification of FAs with CoA drives FA uptake by a partially defined mechanism. We validated the transport function of FATP1 in RPE by showing that uptake of a fluorescent FA analog uptake was elevated and accelerated in hFATP1TG mice compared with WT mice. Although RPE expresses several a*t*RE synthases, the dominant enzyme is LRAT, which transfers the sn-1 palmitate of lecithin molecules to retinol [4]. Recent work has suggested that diacylglycerol O-acyltransferase-1 (DGAT1) can also synthesize a*t*RE in both the retina and RPE by acting as an acyl-CoA: retinol acyltransferase using palmitoyl-CoA as the acyl donor [30]. Together, LRAT and DGAT1 may exploit the increased FA uptake in hFATP1TG RPE to promote *at*RE synthesis. We previously showed *in vitro* that hFATP1 alters the balance of a*t*RE synthesis, suggesting that it might stimulate reversal of the LRAT reaction [9]. However, we observed no compensatory change in Lrat (S2 Fig) or Dgat1 (not shown) expression in hFATP1TG mice compared with WT mice. Therefore, the present data suggest that the ACS activity of FATP1 is not crucial for reversal of the Lrat reaction *in vivo*, but rather, facilitates FA transport and accumulation of a*t*RE.

The excess of *at*RE in hFATP1TG mice was consistent with our two-photon microscopy analysis, which allowed monitoring the intrinsic AF of atRE described within retinosomes or RESTs [31]. RESTs actively participate in 11*c*RAL regeneration in mice, as previously demonstrated [32]. RESTs also accumulate in mice deficient in RPE65 isomerase activity. Thus, the accumulation of REST observed in hFATP1TG mice may be consistent with an inhibition of isomerase activity. These findings suggest a common mechanism for lipid droplet formation and *at*RE storage involving FATP1 at endoplasmic reticulum membranes in RPE cells.

The high levels of a*t*RAL in aged hFATP1TG mice compared with WT mice could be explained by an indirect effect in the POS. Indeed, we showed an increase in rhodopsin expression that correlated with the rise in PR number and POS length. Because of the close anatomical and metabolic relationship between RPE and PRs, we concluded that this could be a non-cell-autonomous effect of hFATP1 overexpression in RPE on PRs. To explain the longer outer segments, we propose that the influx of FAs increases biosynthesis of phospholipids, the main constituents of PR disk membranes. These membranes contain thousands of rhodopsin molecules that contribute to the isomerization of 11*c*RAL to a*t*RAL upon activation by photons. AtRAL is released once rhodopsin is inactivated. Thus, it is conceivable that the increase in a*t*RAL was directly related to the increase in rhodopsin. Free a*t*RAL is chemically toxic and can induce oxidative and carbonyl stress [33,34]. Consistent with this, we found that light-induced PR degeneration was markedly increased in the hFATP1TG mice. At the cytosolic disc surface, a*t*RAL is reversibly reduced to a*t*ROL by Rdh8/Rdh12 [35]. A delay in a*t*RAL reduction might also be a consequence of changes in lipid metabolism. For example, alterations in the unique FA composition of disk membranes may affect membrane permeability, fluidity, and size, as well as activation of membrane-bound proteins such as rhodopsin and Abca4 [36,37]. In this study, the ERG response latency was unchanged in hFATP1TG mice, suggesting that the dynamics of rhodopsin activation in response to light absorption were preserved. A number of studies have demonstrated that the a*t*RAL reduction step determines the kinetics of retinol formation [38,39], prompting us to examine *at*Rdh activity. However, we found no difference in the activity in hFATP1TG and WT retinas (S3 Fig). Therefore, the mechanism governing the accumulation of a*t*RAL with age remains unclear and requires further investigation.

Remarkably, expression of the hFATP1 transgene increased the number and length of PRs in this mouse model, revealing a new function for FATP1 during retinal development that occurs, at least partially, via the PR apoptotic wave. FATP1-mediated uptake of FAs, including long chain polyunsaturated FAs, is thought to play a protective role in neurological disorders in the adult and to be involved in the etiology of metabolic pathologies such as cardiovascular and immune diseases, diabetes, and obesity [40,41]. Docosahexaenoic acid (DHA), the most abundant long chain polyunsaturated FA in the brain and retina, contributes to the formation of membrane phospholipids that play an essential part in normal retinal and visual function in humans, especially early in postnatal life [42,43]. Ochiai et al. [44] recently reported that FATP1 contributes to the transport of DHA into the brain. DHA suppression of apoptosis can be partially attributed to changes in membrane composition. In neurons, incorporation of DHA into phospholipids promotes signaling via the phosphoinositide 3-kinase (PI3K)/AKT pathway and prevents apoptosis [45]. In rods, PI3K activity is regulated by light; thus, light-induced activation of PI3K may initiate an innate self-protective mechanism [46]. Alternatively, neuroprotective oxygenated products of DHA might prevent apoptosis by activating antiapoptotic Bcl-2 and inhibiting proapoptotic BAX [47]. We showed here that mice Bcl-2 and Bax mRNAs are upregulated and downregulated, respectively, by hFATP1 overexpression. Likewise, Qi et al. [25] demonstrated that FATP1 silencing significantly increased the expression and activities of the proapoptotic factors caspase 3 and Bax. Interestingly, in *Drosophila*, *fatp* deficiency causes loss of PRs via a caspase-dependent death pathway, suggesting an important conserved function for FATP in PR survival [18]. Surprinsingly, the increased number of PRs and their longer outer segments did not cause a change in the ERG responses. Some explanations could be proposed to limit sensitivity to light: 1) enlargement of the PR layer occurred only in discrete regions of the retina, 2) the 11*c*RAL rate did not change in hFATP1TG retina, 3) accumulation of a*t*RAL, and 4) potential change in POS membrane lipid composition.

We [14] and others [11] have previously studied the roles of FATP1 and FATP4 in visual function. FATP1-deficient mice showed a delayed recovery of the b-wave amplitude after bleaching but the visual cycle kinetics were unchanged, suggesting that FATP1 was required for PR function and that FATP4 might compensate for the loss of isomerase activity in the visual cycle. Consistent with these results, FATP4 was recently reported to inhibit RPE65 isomerase, and FATP4-deficient mice display faster regeneration of 11*c*RAL. In conclusion our present study demonstrates that overexpression of FATP1 may be relevant to the isomerase inhibition and identify a previously unappreciated role for FATP1 as an important regulator of retinoid metabolism and photoreceptor homeostasis.

## Supporting information

S1 Fig Fatp4 expression is unchanged in hFATP1TG mice. QPCR analysis of mouse Fatp4 mRNA in hFATP1TG and wild type (WT) mice. mFatp4 mRNA in the RPE-choroid (n = 15) and neural retina (n = 10) is normalized to Mertk and actin mRNA, respectively.
(TIF)

S2 Fig Expression of visual cycle enzymes in the RPE of hFATP1TG and wild type mice. (A) qPCR analysis of RPE65, lecithin retinol acyltransferase (LRAT), and 11-*cis*-retinal dehydrogenase (RDH5) mRNA in RPE-choroid of hFATP1TG and wild type (WT) mice. mRNA levels were normalized to Mertk (n = 15). (B) Western blots of RPE65 (left) and LRAT and RDH5 (right) protein in RPE-choroid of hFATP1TG and WT mice. Tubulin and actin served as loading controls.
(TIF)

S3 Fig Retinal dehydrogenase activity in the neural retina of hFATP1TG and wild type mice. A*t*Rdh enzymatic activity was measured by quantification of *at*ROL formation in NR homogenates from hFATP1TG and WT mice, n = 3 mice per time point.
(TIF)
