# Supplementary figures and images for: Fatty acid transport protein 1 regulates retinoid metabolism and photoreceptor development in mouse retina
Source: PLoS One. 2017 Jul 3;12(7):e0180148. doi: 10.1371/journal.pone.0180148 (PMC5495297; doi:10.1371/journal.pone.0180148)

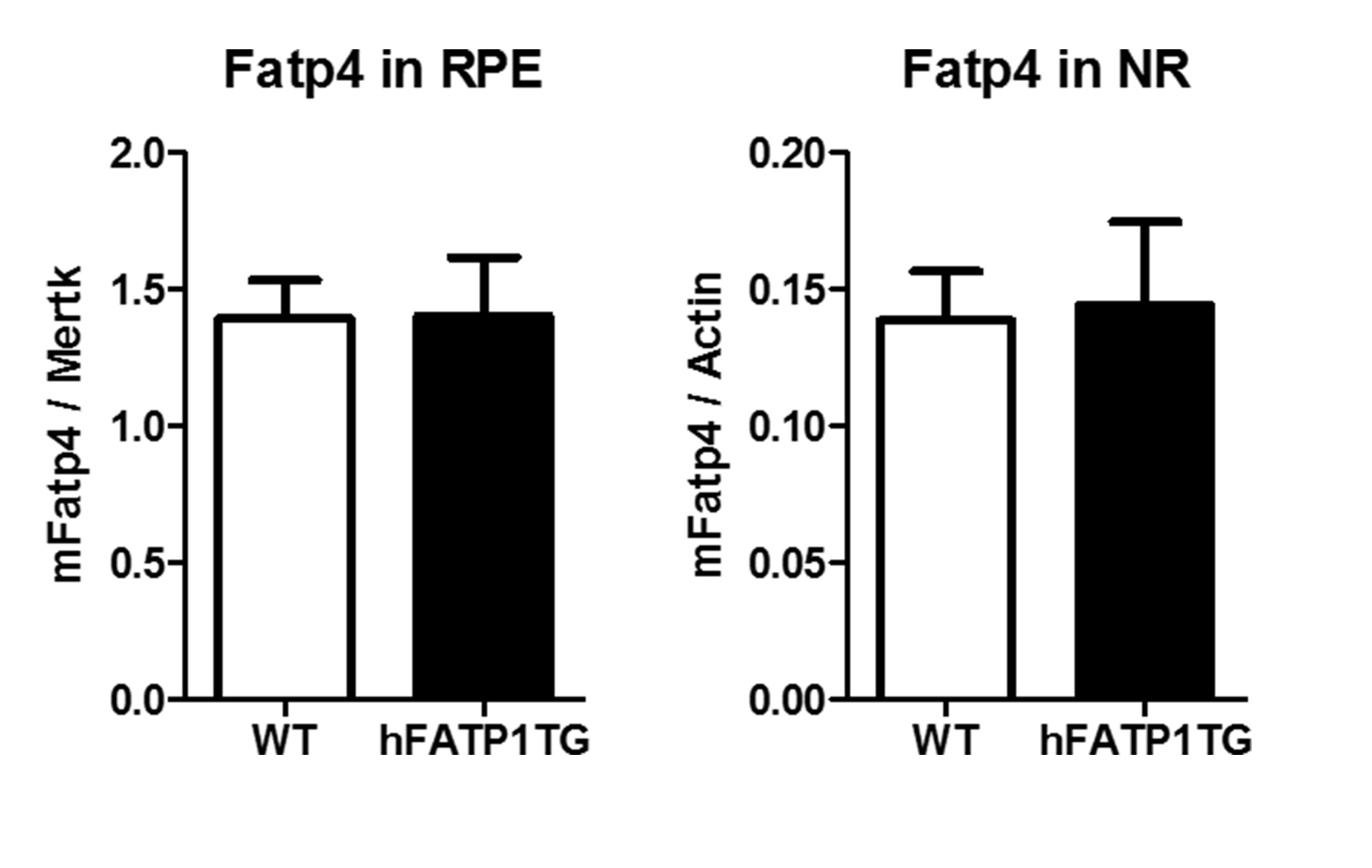

Supplement: S1 Fig — QPCR analysis of mouse Fatp4 mRNA in hFATP1TG and wild type (WT) mice. mFatp4 mRNA in the RPE-choroid (n = 15) and neural retina (n = 10) is normalized to Mertk and actin mRNA, respectively. (TIF) [file pone.0180148.s001.tif]

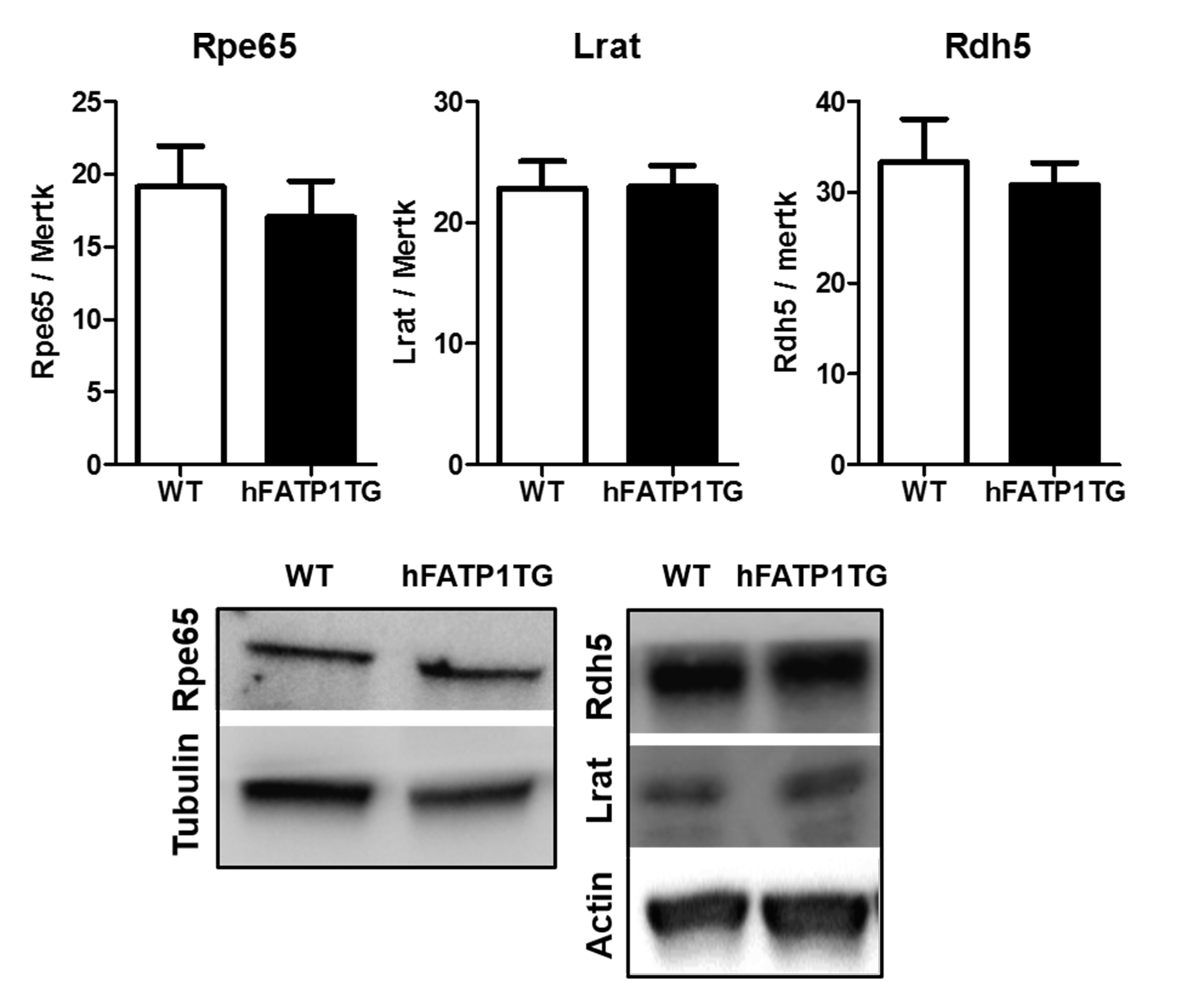

Supplement: S2 Fig — (A) qPCR analysis of RPE65, lecithin retinol acyltransferase (LRAT), and 11-cis-retinal dehydrogenase (RDH5) mRNA in RPE-choroid of hFATP1TG and wild type (WT) mice. mRNA levels were normalized to Mertk (n = 15). (B) Western blots of RPE65 (left) and LRAT and RDH5 (right) protein in RPE-choroid of hFATP1TG and WT mice. Tubulin and actin served as loading controls. (TIF) [file pone.0180148.s002.tif]

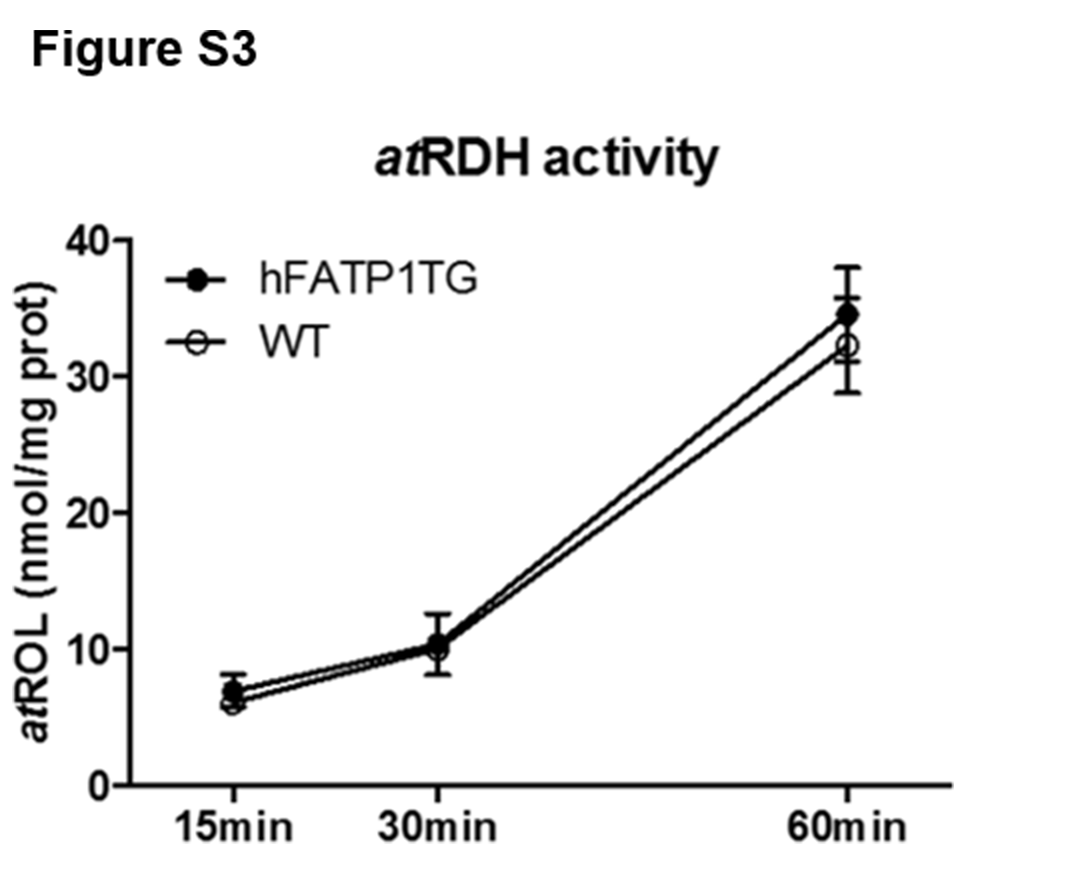

Supplement: S3 Fig — AtRdh enzymatic activity was measured by quantification of atROL formation in NR homogenates from hFATP1TG and WT mice, n = 3 mice per time point. (TIF) [file pone.0180148.s003.tif]
